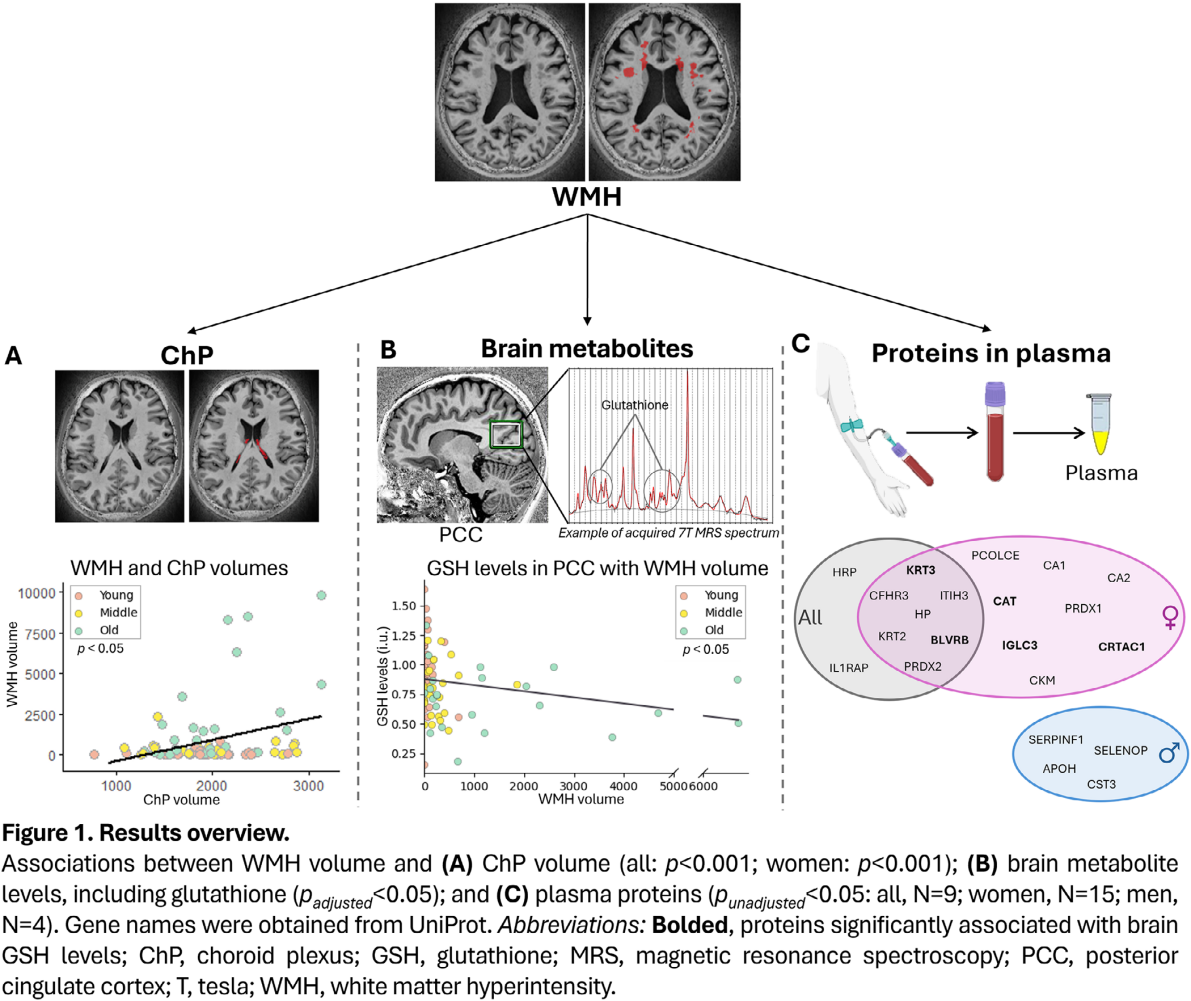# White matter hyperintensities are associated with glymphatic impairment and antioxidant pathway in healthy aging

**DOI:** 10.1002/alz70856_106640

**Published:** 2026-01-08

**Authors:** Flavie E. Detcheverry, Fanta Dabo, Manpreet Singh, Alexandra T. Star, Sneha Senthil, Soraya Lahlou, Ali Filali‐Mouhim, Rozie Arnaoutelis, Dumitru Fetco, Jamie Near, Arsalan S. Haqqani, Sridar Narayanan, AmanPreet Badhwar

**Affiliations:** ^1^ University of Montreal, Montreal, QC, Canada; ^2^ Multiomics Investigation of Neurodegenerative Diseases (MIND) Laboratory, Montreal, QC, Canada; ^3^ Centre de recherche de l'Institut universitaire de geriatrie de Montreal (CRIUGM), Montreal, QC, Canada; ^4^ Institute of Biomedical Engineering, University of Montreal, Montreal, QC, Canada; ^5^ Human Health Therapeutics Research Centre, National Research Council, Ottawa, ON, Canada; ^6^ McConnell Brain Imaging Centre, Montreal Neurological Institute, McGill University, Montreal, QC, Canada; ^7^ University of Toronto, Toronto, ON, Canada; ^8^ Sunnybrook Research Institute, Toronto, ON, Canada

## Abstract

**Background:**

White matter hyperintensities (WMHs), a marker of vascular‐brain injury in older adults, constitute an Alzheimer's disease (AD) risk‐factor (Debette *et al.*, 2019). Recent studies in AD associate WMHs to (a) enlarged choroid plexus (ChP) (Hong *et al.*, 2024), a structure involved in glymphatic clearance of AD‐related proteins and waste from the brain (Hauglund *et al.*, 2020), and (b) lower brain levels of glutathione, an antioxidant metabolite (Detcheverry *et al.*, 2024). However, the interplay between WMHs, ChP, and brain metabolites in healthy aging remains poorly understood, which is essential for unraveling their role in dementia progression. We investigated the relationship between WMH volume, ChP volume, brain metabolites, and plasma proteins.

**Method:**

7T‐MRI/MRS and plasma samples were acquired from 83 healthy adults (42W/41M) aged 20‐79. ChP and WMHs were manually segmented on T1‐weighted MP2RAGE and 3D FLAIR images, respectively, and normalized for head size. Metabolites in the posterior cingulate cortex were measured with single‐voxel STEAM MRS. Mass spectrometry‐based proteomics was performed on plasma. GLMs were performed for WMH volume and ChP volume (controlling for age); and MRS‐detected brain metabolites. Exploratory analyses were performed on plasma proteins and (a) WMH volume (controlling for age), followed by functional enrichment analysis on the significant proteins using STRING; and (b) MRS‐detected brain metabolites (Spearman).

**Result:**

We found significant associations (Figure 1) between WMH volume and (a) ChP volume (all: *p* <0.001; women: *p* <0.001), (b) five brain metabolites, including glutathione (*p_adjusted_<*0.05), and (c) age (all: *p* <0.001; women: *p* <0.05; men: *p* <0.05). WMH volume was also significantly associated with 21/315 plasma proteins (*p_unadjusted_* <0.05: all, *N* = 9; women, *N* = 15; men, *N* = 4), with the majority of biological processes linked to antioxidant activity (*p_adjusted_<*0.05). Additionally, several of the 21 proteins (e.g., flavin reductase [NADPH], catalase) were significantly associated (*p_unadjusted_* <0.05) with brain levels of the antioxidant glutathione.

**Conclusion:**

We demonstrated a link between greater WMH and ChP volumes, indicating an interplay between vascular‐brain injury and glymphatic function, with the antioxidant system playing a key role.